# Cottage Benefit Nursing Association

**Published:** 1919-11-01

**Authors:** 


### Cottage Benefit Nursing Association.

In view of the mention of this Association in the letter signed "L. E. G.," published in our correspondence columns this week, our readers may be interested to have the following up-to-date information. The Association has its training centre in London, and provides a course of twelve months' tuition in theoretical and practical work in all nursing subjects, including midwifery and the C.M.B. qualification, to women desiring to train as cottage nurses. Candidates are accepted for twelve months' training at the age of twenty-three, or at a younger age for fifteen months' training, including maternity nursing, but not midwifery. All receive a salary of £10 while training, after which they are expected to sign an agreement to work for the Association not less than three years at a salary of £20, rising to £25 and £30 in the second and third years respectively, with a bonus of six guineas on completion of three years' satisfactory service. Full outdoor and part indoor uniform, board, lodging, laundry, and a bag of nursing requisites are provided each candidate. Two weeks' holiday are given after training, and two to four weeks in each year following. A certificate is granted on successfully passing the examination at the end of the first year, but is held by the central office until the termination of three years' service. When qualified the nurse proceeds to a branch in a country district, and when on duty lives at the home of her patient. Where no domestic help is provided she is expected to do all that is required for the family except very heavy or rough work.

